# Association between dietary niacin intake and the odds of gallstones in US adults: A cross-sectional study in NHANES 2017–2020

**DOI:** 10.1016/j.pmedr.2025.103057

**Published:** 2025-04-10

**Authors:** Huadi Chen, Wenting Zhao, Yi Xiao, Qiaoping Gao, Xiaoqu Yang, Kangfeng Pang, Baoyi Huang, Xiaolu Liang

**Affiliations:** a Department of Hepatobiliary Surgery, Affiliated Hospital of Guangdong Medical University, Zhanjiang, Guangdong Province, People's Republic of China; b Development Planning Department, Affiliated Hospital of Guangdong Medical University, Zhanjiang, Guangdong Province, People's Republic of China; c Department of Breast Surgery, Affiliated Hospital of Guangdong Medical University, Zhanjiang, Guangdong Province, People's Republic of China

**Keywords:** Dietary intake, Niacin, Gallstones, NHANES, Cross-sectional study

## Abstract

**Objective:**

To investigate the association between dietary niacin intake and the risk of gallstones in American adults using data from the National Health and Nutrition Examination Survey (NHANES) from 2017 to 2020.

**Methods:**

This cross-sectional study analyzed data from 8191 participants aged 18 years and older. Dietary niacin intake was assessed using two 24-h dietary recalls. The presence of gallstones was identified through a questionnaire. Logistic regression models were used to estimate odds ratios (ORs) and 95 % confidence intervals (CIs) for gallstones across quartiles of niacin intake, adjusting for demographic and health-related covariates.

**Results:**

Participants with higher niacin intake showed a significantly lower risk of gallstones. After adjusting for a wide range of covariates, individuals in the highest quartile of niacin intake had a 49 % reduced risk of gallstones compared to those in the lowest quartile (OR = 0.51, 95 % CI: 0.34, 0.76).

**Conclusion:**

Higher dietary niacin intake is associated with a reduced risk of gallstones in US adults. These findings suggest that increasing niacin intake could be a viable strategy for the prevention of gallstones. Future longitudinal studies are needed to confirm these results and explore the underlying mechanisms.

## Highlights

• Higher dietary niacin intake is linked to a lower risk of developing gallstones.
• A restricted cubic spline analysis confirmed the inverse trend of niacin and gallstones.
• Findings suggest that increasing niacin-rich foods may help reduce gallstone formation.

## 1 Introduction

Gallstones, or cholelithiasis, are crystalline concretions formed within the gallbladder by accretion of bile components. These stones can vary greatly in size and can primarily consist of hardened cholesterol, bilirubin, and calcium salts. While often asymptomatic, gallstones can cause biliary colic—a condition marked by severe abdominal pain, nausea, and vomiting—if they obstruct a bile duct, sometimes requiring surgical intervention (Lammert et al., 2016; EASL Clinical Practice Guidelines on the prevention, 2016).

In the United States, the prevalence of gallstones has been increasing over time. Recent studies estimate that it affects approximately 13.9 % of the adult population, with rates rising particularly among women, Hispanics, and older adults (Unalp-Arida and Ruhl, 2023; Unalp-Arida and Ruhl, 2022).This equates to millions of individuals who bear the clinical and economic burdens associated with this condition (Unalp-Arida and Ruhl, 2022; Shaffer, 2005). Surgical removal of the gallbladder remains the primary treatment for symptomatic cases, though medications and lifestyle changes may provide relief (Lammert et al., 2016).

Niacin, also known as vitamin B3, is an essential nutrient found in various animal-based and plant-based foods. It serves as a key component of the coenzymes nicotinamide adenine dinucleotide and nicotinamide adenine dinucleotide phosphate, which support metabolic processes that convert macronutrients into energy (Makarov et al., 2019; Sauve, 2008; MacKay et al., 2012). Furthermore, niacin plays a significant role in DNA repair and synthesis (Surjana et al., 2010), helping to maintain cellular health and function. Its antioxidant properties assist in reducing oxidative stress and supporting immune function (Feng et al., 2016). Niacin also contributes to cardiovascular health by helping to manage cholesterol levels, specifically by lowering harmful LDL cholesterol and triglycerides while increasing beneficial HDL cholesterol (D'Andrea et al., 2019).

While considerable research has been done on the impact of overall diet on gallstone risk, the specific contributions of individual nutrients like niacin have been less thoroughly examined. This is despite preliminary evidence suggesting that niacin's metabolic roles could influence key risk factors for gallstone formation, such as cholesterol levels and liver health. The hypothesized mechanism by which niacin intake may reduce gallstone risk primarily involves its effect on lipid metabolism. Niacin has been shown to decrease serum cholesterol and triglyceride levels (Ganji et al., 2003), which could help prevent cholesterol from supersaturating the bile—a known precipitating factor for cholesterol gallstone formation. Moreover, niacin's role in enhancing liver function and promoting bile flow might also mitigate gallstone risk. The need to explore the relationship between niacin intake and gallstones is underscored by the potential impact of dietary factors on the prevalence and management of gallstones. Understanding this relationship could inform dietary strategies to decrease the incidence of gallstones and reduce the need for surgical interventions.

This study aims to investigate the association between dietary niacin intake and the risk of gallstone development using a comprehensive dataset from the National Health and Nutrition Examination Survey (NHANES) from the years 2017 to 2020. Identifying dietary factors linked to gallstones may provide insights for preventive strategies.

## 2 Methods

### 2.1 Study design and population

**Fig. 1:**
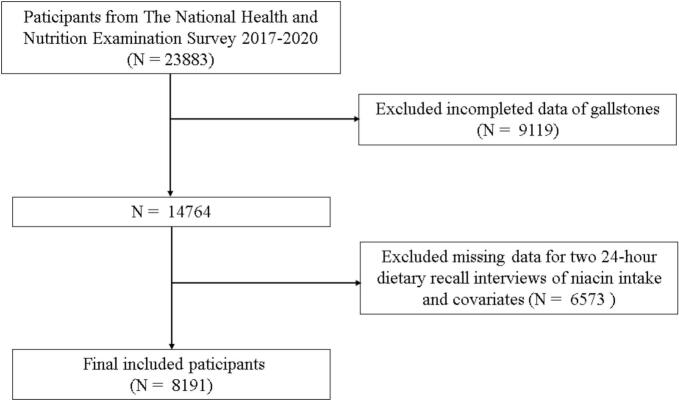
Flowchart of participant selection from National Health and Nutrition Examination Survey (NHANES 2017–2020) for the analysis of association between dietary niacin intake and the odds of gallstones.

This study is based on the NHANES database, which is publicly available and accessible at https://www.cdc.gov/nchs/nhanes/index.htm. All NHANES study protocols were approved by the National Center for Health Statistics Ethics Review Board. Written informed consent was obtained from all participants prior to data collection. Since the data are fully anonymized and publicly accessible, additional Institutional Review Board approval was not required for this secondary analysis. At no point during the study did we have access to information that could identify individual participants. This cross-sectional study utilized data from NHANES collected between 2017 and 2020. We included participants aged 18 years and older who had complete data incuding common demographic characteristics, dietary niacin intake and gallstones status. The study initially enrolled 23,883 participants. Of these, 9119 provided indeterminate responses to the gallstone questionnaire, leaving 14,764 participants who fully completed the assessment. Among these, only 8191 participants had complete data on relevant characteristics and dietary niacin intake. A flow chart illustrating this selection process is shown in Fig. 1.

### 2.2 Dietary Assessment and identification of covariates

In our study, dietary data were collected using the U.S. Department of Agriculture's (USDA) automated multiple pass method, which NHANES employs to conduct two 24-h dietary recalls. The initial recall was conducted face-to-face in a mobile examination center, followed by a second recall via a telephone interview, typically conducted 3 to 10 days after the first. Nutrient intakes are calculated using the USDA's Food and Nutrient Database for Dietary Studies (FNDDS version 2.0), with comprehensive guidelines provided in Appendixes. This dataset provided comprehensive records of nutrient intake per individual. For the purposes of our analysis, we calculated daily niacin intake by averaging the two dietary recall results for each participant. Our statistical model was designed to control for various factors that could influence the study outcomes. These covariates included age, sex, race/ethnicity, education level, Body Mass Index (BMI), dietary energy intake, presence of hyperlipidemia, cardiovascular disease, smoking status, alcohol consumption, and histories of diabetes and hypertension.

### 2.3 Outcome measures

According to the previous report (Wang et al., 2023), the primary outcome of the study was the presence of gallstones, identified by questionnaire that asked respondents, “Has a doctor or other health professional ever told you that you had gallstones?”. Participants were categorized based on the presence or absence of gallstones.

### 2.4 Statistical analysis

Continuous variables in the study were expressed as means ± standard deviation (SD), whereas categorical variables were described using frequencies (percentages). To explore differences between groups, continuous variables were analyzed using Student's *t*-tests, and categorical variables were assessed using Chi-square tests. Dietary niacin intake levels were divided into quartiles based on the distribution within the study population. The boundaries of these quartiles were established by arranging the daily niacin intake of all participants from lowest to highest and then dividing the data into four equal groups, ensuring an even distribution of participants across each quartile. This method provides a clear stratification based on intake levels, allowing for comparative analysis across groups. Logistic regression analyses were conducted to compute odds ratios (ORs) and 95 % confidence intervals (CIs) across quartiles of niacin intake in relation to gallstone incidence. Two distinct logistic regression models were developed: Model 1: A crude model with no covariate adjustments; Model 2: This comprehensive model included adjustments for age, sex, race/ethnicity, education level, BMI, dietary energy intake, hyperlipidemia, cardiovascular disease, smoking status, alcohol consumption, and histories of diabetes and hypertension, providing a robust analysis of the factors influencing gallstone formation. We selected the fully adjusted model (Model 2) as the primary analysis to control for potential confounding factors comprehensively. The unadjusted model (Model 1) is presented for comparison.

Additionally, a restricted cubic spline analysis was conducted to further examine the relationship between dietary niacin intake and gallstone risk, adjusting for all listed covariates. All statistical tests were two-sided, and a *p*-value <0.05 was considered statistically significant. To ensure the representativeness and accuracy of our findings from the NHANES data, all statistical analyses incorporated the complex survey design by applying appropriate sample weights, strata, and primary sampling units. Statistical analyses were conducted using R software (version 4.3.0), employing the ‘survey’ package to appropriately apply the NHANES weighting and variance estimation techniques.

## 3 Results

### 3.1 Baseline characteristics of the study participants

In this study, the baseline characteristics were compared between participants with and without gallstones (Table 1). Participants without gallstones exhibited a higher average daily niacin intake (26.51 mg ± 0.32) compared to those diagnosed with gallstones (22.35 mg ± 0.56), with statistical significance (*p* < 0.001). The age and BMI of participants also differed significantly between groups. Those with gallstones were older (average age 55.15 years ±0.78 vs. 46.05 years ±0.49) and had a higher BMI (33.15 kg/m^2^ ± 0.40 vs. 29.37 kg/m^2^ ± 0.17) than their counterparts without the disease, with both differences being highly significant (*p* < 0.0001). The sex distribution showed a higher proportion of females in the gallstones group compared to the non-affected group, and this difference was statistically significant (p < 0.0001).

**Table 1:** Baseline characteristics of study participants with and without gallstones in NHANES (2017–2020).

Variable	Gallstones		*P* value
	No	Yes	
Niacin intake, mg	26.51 ± 0.32	22.35 ± 0.56	< 0.01⁎
Age, years	46.05 ± 0.49	55.15 ± 0.78	< 0.01⁎
BMI, kg/m^2^	29.37 ± 0.17	33.15 ± 0.40	< 0.01⁎
Energy intake, kcal	2116.65 ± 16.77	2029.25 ± 50.03	0.1⁎

Sex, n (%)			< 0.01^#^
female	3656 (85.99)	592 (14.01)	
male	3722 (95.25)	221 (4.75)	

Race/ethnicity, n (%)			0.13^#^
black	2010 (93.85)	155 (6.15)	
Mexican American	860 (89.65)	108 (10.35)	
white	2564 (89.98)	352 (10.02)	
other	1944 (90.46)	198 (9.54)	

Education, n (%)			0.11^#^
college	4620 (91.03)	498 (8.97)	
less than college	2758 (89.35)	315 (10.65)	

Hyperlipidemia, n (%)			< 0.01^#^
no	2654 (93.06)	204 (6.94)	
yes	4724 (89.01)	609 (10.99)	

Hypertension, n (%)			< 0.01^#^
no	4606 (93.01)	381 (6.99)	
yes	2772 (85.60)	432 (14.40)	

Diabetes, n (%)			< 0.01^#^
no	6120 (91.58)	566 (8.42)	
yes	1258 (83.61)	247 (16.39)	

Cardiovascular disease, n (%)			< 0.01^#^
no	6708 (91.17)	671 (8.83)	
yes	670 (82.58)	142 (17.42)	

Smoke, n (%)			0.02^#^
former	1621 (88.23)	238 (11.77)	
now	1361 (90.82)	149 (9.18)	
never	4396 (91.23)	426 (8.77)	

Alcohol user, n (%)			0.07^#^
heavy	1610 (92.65)	145 (7.35)	
moderate	1543 (89.61)	186 (10.39)	
mild	3393 (90.08)	385 (9.92)	
never	832 (88.87)	97 (11.13)	

Data was expressed as means ±SD or n(%).

BMI, body mass index; NHANES, National Health and Nutrition Examination Survey;

SD, standard deviation; n (%), frequencies (percentages).

⁎ t-test; # chi-square.

Furthermore, there were no significant differences in racial composition or educational levels between the two groups, indicating that these factors did not correlate significantly with the presence of cholelithiasis. Health conditions such as hyperlipidemia, hypertension, diabetes, and cardiovascular diseases were more prevalent in participants with cholelithiasis, highlighting a significant association with these comorbidities (all *p*-values <0.0001). Lifestyle factors, including smoking status and alcohol consumption showed variations, but these did not uniformly reach statistical significance, suggesting a complex interaction with gallstone risk. These comprehensive demographic and health profiles underline the multifaceted nature of gallstones risk factors and support the need for targeted interventions based on individual health profiles.

### 3.2 Association between dietary niacin intake and the risk of gallstones

Table 2 details the relationship between dietary niacin intake and the risk of developing gallstones, analyzed by a segmentation of participants into quartiles based on their levels of niacin consumption. The first quartile, representing the lowest level of niacin intake, served as the control group.

**Table 2:** The odds ratio for dose-response relationship between dietary niacin intake and gallstones in NHANES 2017–2020.

	Dietary niacin intake, mg				p for trend
	Q1, [2.42, 16.36]	Q2, [16.36, 22.50]	Q3, [22.50, 30.19]	Q4, [30.19, 171.35]	
Model 1	1.00	0.56 (0.43, 0.72)	0.47 (0.33, 0.67)	0.43 (0.29, 0.63)	< 0.01
Model 2	1.00	0.56 (0.42, 0.76)	0.51 (0.34, 0.76)	0.51 (0.34, 0.76)	< 0.01

Data was shown as OR (95 % CI).

Model 1: crude model.

Model 2: adjusted for age, sex, race/ethnicity, educational level, energy intake, BMI, hypertension, hyperlipidemia, diabetes, cardiovascular disease, smoking, alcohol consumption.

Q, Quartile; OR, odds ratio; CI, confidence interval; BMI, body mass index; NHANES, National Health and Nutrition Examination Survey.

**Fig. 2:**
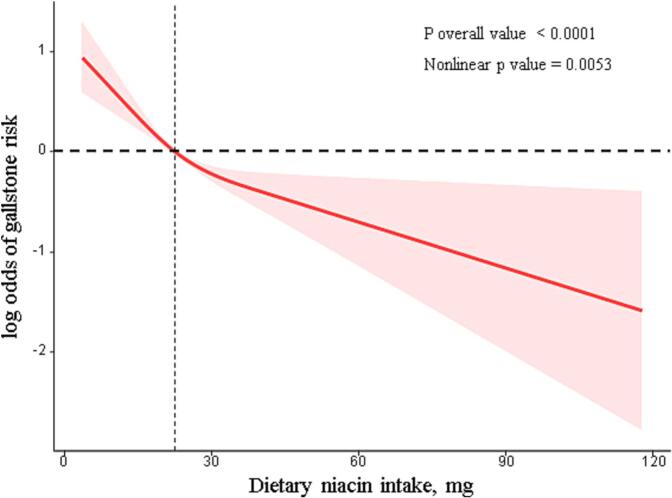
Dose–response relationship between dietary niacin intake and gallstone risk based on National Health and Nutrition Examination Survey (NHANES 2017–2020). Restricted cubic spline analysis was performed to evaluate the association between dietary niacin intake and the risk of gallstones. The solid line represents the adjusted odds ratio, and the shaded area indicates the 95 % confidence interval. The model was adjusted for age, sex, race/ethnicity, educational attainment, body mass index (BMI), total dietary energy intake, hyperlipidemia, cardiovascular disease, smoking status, alcohol use, and prior diagnoses of diabetes and hypertension.

In the initial analysis (Model 1), which did not adjust for any variables, a clear inverse correlation was observed between niacin intake and the risk of gallstones. Specifically, individuals in the second, third, and fourth quartiles experienced a respective 44 %, 53 %, and 59 % reduction in gallstone risk compared to the baseline group (OR = 0.56, 95 % CI: 0.43, 0.72; OR = 0.47, 95 % CI: 0.33, 0.67; OR = 0.43, 95 % CI: 0.29, 0.63). Thecomprehensive analysis, Model 2, adjusted for a wide range of covariates such as age, sex, race/ethnicity, educational level, BMI, dietary energy intake, hyperlipidemia, cardiovascular disease status, smoking habits, alcohol consumption, and medical histories of diabetes and hypertension. In this model, the second, third, and fourth quartiles all exhibited a consistent 44 %, 49 %, and 49 % lower risk of developing gallstones compared to the first quartile (OR = 0.56, 95 % CI: 0.42, 0.76; OR = 0.51, 95 % CI: 0.34, 0.76; OR = 0.51, 95 % CI: 0.34, 0.76), demonstrating a strong inverse association. Additionally, a restricted cubic spline analysis further supported these findings, revealing a significant nonlinear inverse relationship between dietary niacin intake and gallstone occurrence (p-nonlinear = 0.0053), as depicted in Fig. 2. This pattern underscores the potential protective effect of higher niacin intake against gallstone formation.

## 4 Discussion

This study reveals a significant inverse association between dietary niacin intake and gallstones risk. Participants with higher niacin intake had significantly lower odds of developing gallstones, an effect that persisted even after adjusting for multiple covariates in Model 2. The robust inverse correlation observed in the restricted cubic spline analysis further supports the theory that increased niacin intake may help prevent the pathogenesis of cholesterol gallstones. These findings confirm the unique role of niacin intake in reducing the risk of gallstones.

The potential protective effect of niacin against gallstone formation can be explained through multiple physiological mechanisms. Firstly, niacin plays a crucial role in lipid metabolism (Blond et al., 2014), effectively reducing serum levels of low-density lipoprotein cholesterol and triglycerides while simultaneously increasing high-density lipoprotein cholesterol. Since cholesterol supersaturation in bile is a key factor in gallstone pathogenesis, niacin's lipid-modulating properties may reduce bile cholesterol saturation and lower the risk of cholesterol crystallization, thereby preventing gallstone formation. Additionally, niacin enhances bile acid secretion, which is essential for maintaining bile fluidity and preventing cholesterol precipitation. A higher bile acid-to-cholesterol ratio contributes to a more stable bile composition (Camilleri and Gores, 2015), reducing the likelihood of gallstone formation. Beyond its effects on bile composition, niacin also exerts anti-inflammatory and antioxidant properties, which help reduce hepatic oxidative stress and chronic inflammation—both of which are associated with impaired bile metabolism and gallstone formation (Ferrell and Chiang, 2019). Furthermore, niacin may influence gallbladder motility, an important factor in preventing bile stasis. Evidence suggests that niacin improves gallbladder contractility, facilitating bile flow and minimizing the accumulation of lithogenic bile that predisposes individuals to stone formation (Park et al., 2022). Another key mechanism involves niacin's role in metabolic regulation, particularly in glucose and insulin homeostasis (Yang et al., 2018). Given that metabolic disorders such as obesity and diabetes are established risk factors for gallstones, niacin's ability to enhance insulin sensitivity and regulate glucose metabolism may provide an additional protective effect against gallstone development. Taken together, these mechanisms suggest that increased dietary niacin intake may positively influence lipid metabolism, bile acid secretion, hepatic function, gallbladder motility, and metabolic regulation—all of which are crucial for reducing gallstone risk.

The relationship between diet and gallstones risk has been a subject of considerable scientific inquiry. Previous studies have primarily focused on broad dietary patterns and common nutrients such as fats, carbohydrates, and certain proteins (Jessri and Rashidkhani, 2015; Jia et al., 2023; Madden et al., 2024). However, the specific role of individual micronutrients like niacin has received comparatively less attention in gallstones research. So far, there have been no specific detailed reports. Our findings provide important new insights by highlighting niacin as a potentially significant protective factor against gallstone formation. The protective role of niacin is particularly compelling in contrast to existing literature, which has primarily focused on the impact of more commonly discussed dietary factors (Kotrotsios et al., 2019). For instance, a study reported by Mendez-Sanchez et al. on the role of dietary fat in gallstone disease suggests a complex relationship dependent on the type of fats consumed (Méndez-Sánchez et al., 2003). Compared to such studies, our research offers a nuanced understanding by suggesting how micronutrients, specifically niacin, play a role in modulating disease risk, potentially through mechanisms affecting cholesterol metabolism and liver function (Linder et al., 2019; Romani et al., 2019). Moreover, the apparent protective effect of niacin against gallstones aligns with emerging research on other vitamins, such as vitamins C, D and E, which have been hypothesized to influence gallstone risk through antioxidant properties that may affect bile chemistry (Walcher et al., 2009; Waniek et al., 2018; Shabanzadeh et al., 2016). Our study extends this line of inquiry by providing empirical support for the inclusion of niacin amongnutrients that could have preventive benefits against cholelithiasis. This novel finding is significant because it opens new avenues for both clinical practice and dietary recommendations. It suggests that increasing the intake of niacin-rich foods or considering niacin supplementation may be a viable strategy for reducing gallstone risk, thus providing a potentially cost-effective and accessible means of managing this prevalent condition. Further studies are needed to explore the biochemical mechanisms through which niacin may influence gallstone pathology and to establish definitive dietary guidelines for prevention.

One strength of this study is the use of a large, nationally representative NHANES dataset, which enhances the generalizability of our findings. Additionally, comprehensive adjustments for demographic and health-related covariates provide a clearer picture of niacin's impact. However, some limitations should be noted. The cross-sectional design prevents causal inference, necessitating further longitudinal studies. Additionally, dietary intake was assessed via 24-h recall, which may not fully capture long-term dietary habits and is subject to recall bias. To mitigate this, our study applied dietary recall on two separate occasions to improve accuracy and estimate habitual intake more reliably.

Our findings suggest that increasing dietary niacin intake could be a beneficial strategy for gallstone prevention. Public health initiatives that encourage dietary modifications to include niacin-rich foods could potentially reduce the prevalence and healthcare burden of gallstones. Clinicians might also consider dietary niacin as part of dietary counseling for patients at risk of gallstones. While this study is based on data from NHANES, which is conducted in the United States, its implications may extend beyond the US population. Gallstone disease is a global health concern, with prevalence rates varying across different regions due to dietary habits, genetic predispositions, and healthcare access. For example, Westernized diets high in processed foods and saturated fats have been associated with higher gallstone risk, whereas populations consuming traditional plant-based or fiber-rich diets tend to have lower prevalence rates. Since niacin is widely available in both plant- and animal-based foods, its potential protective role against gallstones may be relevant across different dietary and cultural contexts. Besides, while our findings suggest that higher dietary niacin intake is associated with a reduced risk of gallstones, it is important to consider the potential risks of excessive niacin intake. Niacin supplementation, particularly at high doses, has been associated with adverse effects such as flushing, liver toxicity, gastrointestinal discomfort, and insulin resistance in susceptible individuals. However, it is essential to distinguish between pharmacological doses of niacin used for lipid management and dietary niacin obtained from food sources. The niacin levels associated with gallstone prevention in this study fall within the range of typical dietary intake and are unlikely to reach harmful levels.

In conclusion, this study provides evidence supporting niacin's protective role against gallstones. Future research should aim to confirm these findings through longitudinal cohort studies and randomized controlled trials to establish a causal relationship between dietary niacin intake and gallstone risk reduction. Given the limitations of self-reported dietary data in cross-sectional studies, future investigations should incorporate biomarker-based assessments of niacin status to improve measurement accuracy. Additionally, mechanistic studies are needed to explore how niacin influences bile composition, lipid metabolism, and gallbladder motility to prevent gallstone formation. Further studies should also examine dose-response relationships to determine optimal niacin intake levels for gallstone prevention. It would be valuable to explore whether genetic variations in lipid metabolism and bile acid synthesis modify the protective effects of niacin. Lastly, interventional trials assessing the impact of niacin supplementation versus dietary sources of niacin on gallstone formation could provide insights for targeted dietary recommendations and public health strategies. These insights could contribute to targeted nutritional interventions to reduce gallstone incidence.

## CRediT authorship contribution statement

**Huadi Chen:** Writing – review & editing, Writing – original draft, Methodology, Funding acquisition, Formal analysis, Data curation, Conceptualization. **Wenting Zhao:** Writing – review & editing, Writing – original draft, Data curation, Conceptualization. **Yi Xiao:** Writing – review & editing, Writing – original draft, Data curation, Conceptualization. **Qiaoping Gao:** Supervision, Software, Data curation, Conceptualization. **Xiaoqu Yang:** Visualization, Validation, Supervision, Software, Methodology. **Kangfeng Pang:** Writing – review & editing, Data curation, Conceptualization. **Baoyi Huang:** Writing – review & editing, Writing – original draft, Supervision, Project administration, Conceptualization. **Xiaolu Liang:** Writing – review & editing, Writing – original draft, Supervision, Formal analysis, Data curation, Conceptualization.

## Ethics approval and consent to participate

The NHANES database is publicly accessible and free of charge. The study's methodology has been approved by the Research Ethics Review Board at the National Center for Health Statistics.

## Funding

This study was supported by the high-level talent research start-up fund of the Affiliated Hospital of Guangdong Medical University (1033Z20230050).

## Declaration of competing interest

The authors declare that they have no known competing financial interests or personal relationships that could have appeared to influence the work reported in this paper.

## Data Availability

The data supporting the findings of this study are available through the NHANES database, accessible at https://www.cdc.gov/nchs/nhanes/index.htm. Relevant data are also included within the manuscript and its supplementary information files. For further information, please contact the corresponding author.

## Appendix A Supplementary data

Supplementary material Image 1
